# Investigation of Industrial Polyurethane Foams Modified with Antimicrobial Copper Nanoparticles

**DOI:** 10.3390/ma9070544

**Published:** 2016-07-07

**Authors:** Maria Chiara Sportelli, Rosaria Anna Picca, Roberto Ronco, Elisabetta Bonerba, Giuseppina Tantillo, Mauro Pollini, Alessandro Sannino, Antonio Valentini, Tommaso R.I. Cataldi, Nicola Cioffi

**Affiliations:** 1 Dipartimento di Chimica, Università Degli Studi di Bari “Aldo Moro”, via E. Orabona 4, Bari (BA) 70126, Italy; maria.sportelli@uniba.it (M.C.S.); rosaria.picca@uniba.it (R.A.P.); robertoronco1@libero.it (R.R.); tommaso.cataldi@uniba.it (T.R.I.C.); 2 Dipartimento di Medicina Veterinaria, Università Degli Studi di Bari “Aldo Moro”, St.da P.le per Casamassima Km 3, Valenzano (BA) 70010, Italy; elisabetta.bonerba@uniba.it (E.B.); giuseppina.tantillo@uniba.it (G.T.); 3 Dipartimento di Ingegneria dell’Innovazione, Università del Salento, via per Monteroni, Lecce (LE) 73100, Italy; mauro.pollini@unisalento.it (M.P.); alessandro.sannino@unisalento.it (A.S.); 4 Dipartimento Interateneo di Fisica, Università Degli Studi di Bari “Aldo Moro”, via Amendola 173, Bari (BA) 70126, Italy; antonio.valentini@uniba.it

**Keywords:** copper nanoparticle, polyurethane foam, ETAAS, XPS, nanoantimicrobials

## Abstract

Antimicrobial copper nanoparticles (CuNPs) were electrosynthetized and applied to the controlled impregnation of industrial polyurethane foams used as padding in the textile production or as filters for air conditioning systems. CuNP-modified materials were investigated and characterized morphologically and spectroscopically, by means of Transmission Electron Microscopy (TEM), and X-ray Photoelectron Spectroscopy (XPS). The release of copper ions in solution was studied by Electro-Thermal Atomic Absorption Spectroscopy (ETAAS). Finally, the antimicrobial activity of freshly prepared, as well as aged samples—stored for two months—was demonstrated towards different target microorganisms.

## 1. Introduction

Application of nanotechnology in the textile industry is practiced with two main purposes. The first one aims at the improvement of the textile performance: fibers modified by nanopowders, or carbon nanotubes (CNTs), generally possess higher mechanical resistance in respect with untreated ones [1]. The second purpose deals with the development of multifunctional textiles, i.e., antimicrobial [2], anti-stain [3], water repellent [4,5], anti-static [6,7,8], and self-cleaning ones [9]. Cotton, linen, silk and wool are well-known natural fibers and padding materials, breathable and resistant [10]. However, they are easily degraded by many microorganisms, like spores, molds and bacteria [11,12]. Special finishes, however, can provide them protection from biodegradation: nanotechnology offers a good range of effective tools to protect fabrics from bacterial contamination [2]. During the last century, the discovery and the growing production of polymer-based artificial fibers and paddings completely eliminated the problems related to biodegradation. Polyurethane foams play a fundamental role in the production of seats in the automotive sector, of filters for the conditioning and treatment of air and water, and so on. However, in all these fields, prevention of bacterial adhesion and/or proliferation is extremely important. Metal and metal oxide nanoparticles (NPs), specifically Cu and its oxides, have been already used for this purpose [13,14]. Natural fiber modification with copper is rather prevalent and different reports are present in literature on this topic, especially for the treatment of cotton fabrics [14]. Most of them deal with their CuO or Cu_2_O functionalization [15,16,17,18,19,20,21,22,23], while the use of elemental Cu is reported less frequently [24,25]. Cu(0), in fact, easily undergoes oxidation processes unless suitable stabilizing agents are used. Nevertheless, modification of artificial polymers by nanostructured Cu is less diffused, and it is generally achieved by adding a copper-containing additive to the polymers during the master batch preparation stage [26,27,28,29]. Silicone fibers [30], polyester [26,31,32], and nylon [33] have been successfully modified by both nanostructured CuO and Cu. Polyurethane (PU) has been modified by different types of inorganic clusters, such as Ag [34,35,36,37,38,39,40,41,42,43,44], CNTs (carbon nanotubes) [45], Zn-Ag bimetallic particles [46], tourmaline [47,48], silica [49] and ZnO [50]. Ag is nowadays one of the preferred additives to confer antimicrobial properties to both natural and synthetic fibers, being well known its antiseptic effect against a wide range of microorganisms. Ag-modified PU foams or fibers are fairly common: in fact, silver is able to confer antimicrobial properties and to improve polymer mechanical and rheological properties [43]. However, to the best of our knowledge, only a few reports are present in the literature about the modification of polyurethanes with antimicrobial CuNPs [51,52,53].

Here we report on polyurethane foams modified with colloidal CuNPs that were electrochemically synthesized by means of the so-called sacrificial anode electrolysis (SAE) technique [54]. Post-production functionalization of industrial polyurethane foams was carried out by their impregnation in diluted CuNP colloids. Samples were morphologically and spectroscopically investigated and characterized. Their antimicrobial activity was tested towards three model microorganisms (*Staphylococcus aureus*, *Escherichia coli* and *Kluyveromyces marxianus*), demonstrating CuNPs capability of strongly inhibiting bacterial growth and proliferation.

## 2. Results and Discussion

### 2.1. Synthesis and Characterization of CuNPs

**Figure 1:**
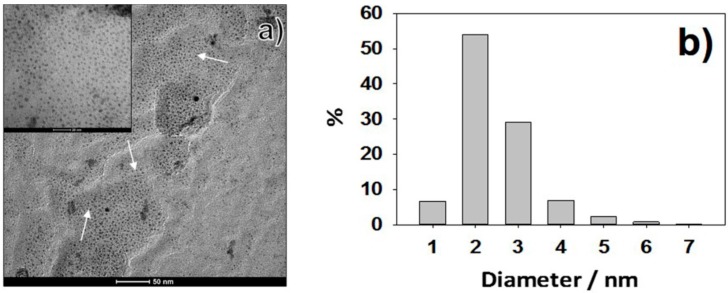
(**a**) Transmission Electron Microscopy (TEM) micrographs of CuNPs (highlighted by arrows) synthesized by sacrificial anode electrolysis. A micrograph at higher magnification is reported as insert; (**b**) Size distribution histogram of as synthesized CuNPs.

CuNPs, used as additives of industrial polyurethane foams, were prepared by sacrificial anode electrolysis as described in the experimental section. Cu concentration in the stock colloidal solution was obtained by differential weighing of working and counter electrodes: it resulted equal to 0.31 ± 0.05 M in as-synthesized Cu-nanocolloids. TOAC (tetraoctylammonium chloride) was chosen as stabilizing agent, due to the high reaction yield of TOAC-driven syntheses (85% ± 5%), excellent morphological control, and lasting storage times of the colloid up to months; there is only the need to avoid air exposure and thermal shocks. Moreover, TOAC octyl chains confer lipophilicity to metal NPs, which become scarcely soluble in water [55]. This attribute can be proficiently used to promote PU impregnation with CuNPs. With regard to pristine Cu-nanocolloids, the high degree of stabilization provided by TOAC capping agent was demonstrated by the TEM micrograph reported in Figure 1a. The electrochemical process resulted in a NP population with a single mode and narrow diameter dispersion, centered at 2.6 nm (σ ≤ 0.5 nm), as shown in the size distribution histogram of Figure 1b.

### 2.2. Adsorption of Industrial Polyurethane Foams with CuNPs

PU samples were treated with CuNP-colloids as reported in Section 3.3. Two different types of polyurethane foam were used. For clarity of presentation, they were labeled as follows:

- Sample A: green foam, with large and irregular pores, used as filling material for mattresses (density: 25 kg/m^3^,density tolerance ±5%);
- Sample B: white foam, with small and regular pores, used in the automotive industry (density: 21 kg/m^3^,density tolerance ±5%).

**Figure 2:**
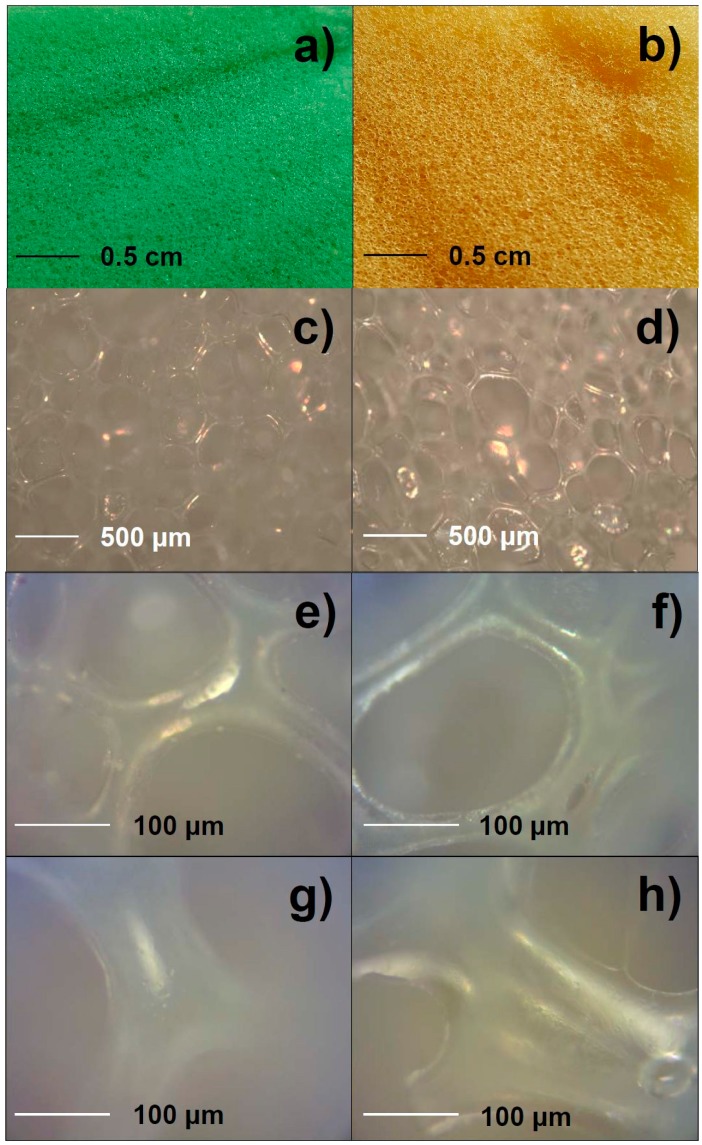
Photographs of (**a**) type A and (**b**) type B pristine polyurethane (PU) foams. Optical micrographs of pristine and Cu-treated foams; (**c**) Low magnification—Pristine type A foam; (**d**) Low magnification—Pristine type B foam; (**e**) High magnification—Pristine type A foam; (**f**) High magnification—Pristine type B foam; (**g**) 1:1000 Cu-modified type A foam; (**h**) 1:1000 Cu-modified type B foam.

Adsorption efficiency was tested by weighing each sample before and after the modification process. Both samples showed an average weight increase equal to 8.2 ± 0.3 mg for type A PU and 7.8 ± 0.2 mg for type B PU, respectively. Pristine and treated foams were subjected to optical microscopy examination. This aimed at ascertaining that the impregnation process did not alter PU pores morphology and/or dimension. Typical optical micrographs are reported in Figure 2. A bigger and more dispersed pore size was observed for pristine samples A (Figure 2a,c,e), while pristine samples B were characterized by a more regular porous structure (Figure 2b,d,f). These features remained unaffected after the impregnation process (Figure 2g,h).

### 2.3. Surface Chemical Characterization of CuNP-Modified Industrial Polyurethane Foams

In order to assess the surface chemical composition of CuNP-modified polyurethane foams, XPS (X-ray photoelectron spectroscopy) analyses were performed. Both A and B PU samples were studied before and after treatment with CuNPs colloids, purposely suspension dilute 100- and 1000-fold with pure solvent. Data of Table 1 show that carbon and oxygen were the most abundant elements on the analyzed surfaces, as expected for an oxygenated polymer dispersing matrix modified by low amounts of CuNPs.

Cu traces could be qualitatively observed on pristine samples, although they were below the XPS limit of quantification. This evidence is reasonable, considering that extrusion of PU foams is performed using copper dyes. Small amounts of Si were also identified: this element is characteristic of PU and PU-based goods production processes [56]. Presence of nitrogen could be attributed to both TOAC (when present) and polymeric backbone. It is also important to point up how Cu-modified samples A had in general a higher Cu atomic %, compared to homologous composites B (e.g., composites obtained by impregnation with the same CuNP dilution). This might be due to the presence of larger pores, which allowed CuNPs to better penetrate into the bulk material. Chlorine is due to the presence of CuNP stabilizing agent (i.e., TOAC), while traces of calcium observed in samples B were considered as a contamination coming from PU industrial production processes.

XP high-resolution (HR) regions were also investigated, in order to obtain detailed information about PU and CuNPs chemical speciation. HR XP C1s spectra of pristine and treated PU foams were made of three main components. As an example, Table 2 resumes data obtained on samples A in terms of peak position (expressed as Binding Energy—BE—values), relative abundance, and signal attribution.

**Figure 3:**
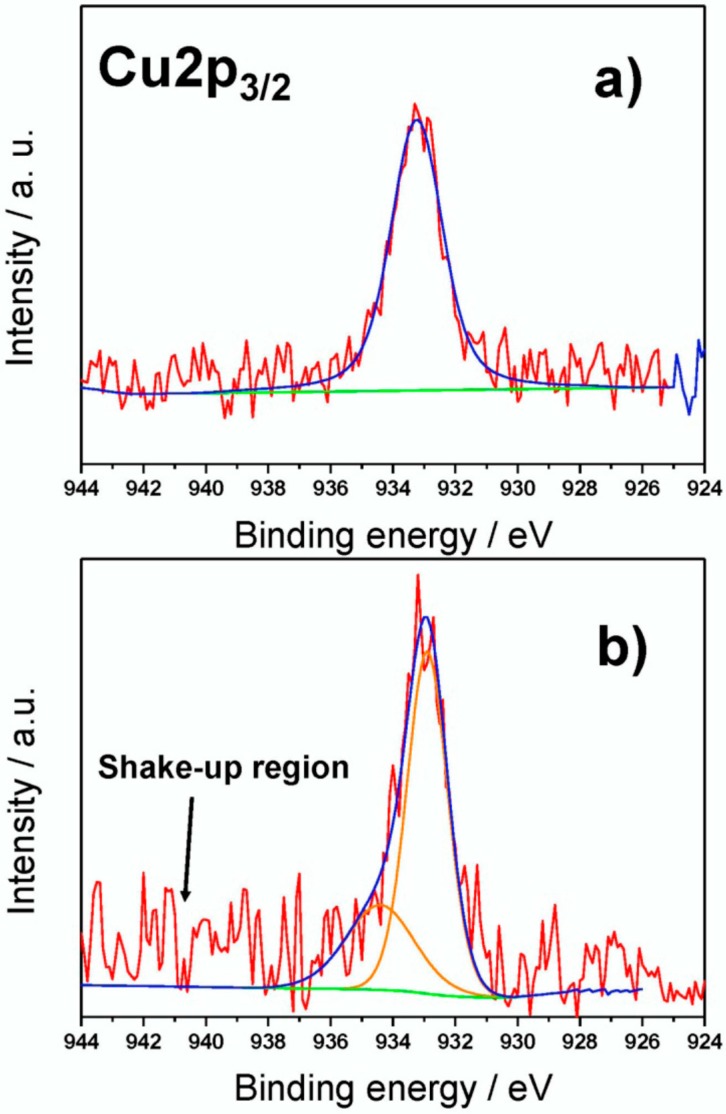
Typical Cu2p_3/2_ X-ray photoelectron (XP) high-resolution spectra of fresh (**a**); and aged (**b**) CuNP-modified polyurethane foams.

All components are in agreement with those expected for PU [57,58]. On Cu-modified foams, a higher relative percentage of aliphatic C–C moieties was observed. This could be explained considering the presence of octyl- alkyl chains from TOAC. HR XP N1s spectra were characterized by a single component in all cases, centered at 400.0 ± 0.2 eV, compatible with PU urethane functionalities [58]. Typical Cu2p_3/2_ XP spectra for fresh and aged samples are reported in Figure 3. Soon after sample preparation (Figure 3a), a single signal falling at 933.2 ± 0.2 eV was present. This peak can be ascribed both to nanodispersed Cu at zero oxidation state [58,59,60] and/or to cuprous species [57,58]. The absence of Cu(II) moieties was confirmed by the lack of photoelectronic signals in the region centered at about 934.0 ± 0.2 eV and the shake-up features between 941.0 and 948.0 eV, as well [57]. CuNPs undergo a certain surface oxidation, as a function of the aging time. This phenomenon led to the formation of cupric moieties onto modified PU foams. Cu2p_3/2_ XP high-resolution region showed, for aged samples (Figure 3b), the presence of a second signal component, at higher binding energy values (934.4 ± 0.3 eV), ascribable to Cu(II) moieties. Shake-up bands were, in this case, barely visible, due to the very high signal-to-noise ratio.

### 2.4. Kinetics of Copper Release from CuNP-Modified Industrial Polyurethane Foams

**Figure 4:**
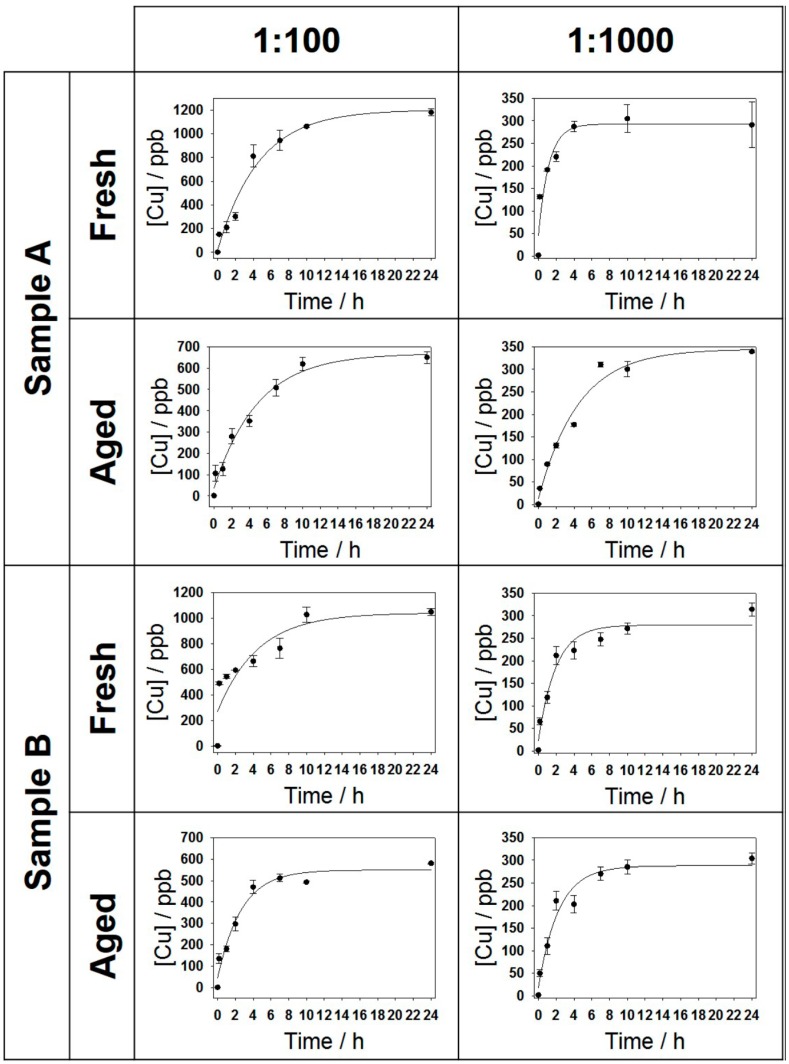
Copper release in physiologic solution from CuNP-modified polyurethane foams, as a function of the incubation time. Different columns are relevant to different CuNP concentrations in the impregnation baths, giving rise to different Cu surface abundance on the composite. Different rows are relevant to composites obtained by different polyurethane batches or to differently aged samples.

CuNP-modified PUs were exposed to a physiological saline solution in order to mimic a possible interaction and ionic release of nanocomposites to model contact media. Experiments were carried out on both freshly prepared and aged samples, which were stored in air for 60 days. The kinetics of Cu release, relative to all the analyzed samples are shown in Figure 4. The experimental data could be interpolated, in all cases, by a pseudo-first order kinetic model.

From the analysis of all the curves, it was found that part of the Cu ion release in solution was quite fast in the first ten minutes. This is reasonably due to the presence of readily soluble Cu species (such as residual cuprous salts) on the surface of CuNPs. It was also evident, in all cases, that Cu concentration progressively grew as a function of time up to a plateau value. The extent of the latter varied according to the concentration of the impregnation bath, and as a function of the aging time, too. In both samples, in fact, a higher mean plateau value was registered for PU foams treated with 1:100 colloidal dilutions, which had the higher surface Cu concentration. The larger pore size of composites based on A-type PU might explain the slightly higher and faster Cu release recorded for materials treated with 1:100 CuNPs dilution, due to a possible easier accessibility of the physiological solution within samples A. In principle, a larger pore size should allow a deeper and easier penetration of physiological solution within the foam. Moreover, for both samples A and B, aging resulted in a considerable decrease of the Cu release. A tentative interpretation of this phenomenon was that surfactant could, over time, segregate [61,62,63] and partially occlude PU pores, enhancing the surface hydrophobicity of the composite and partially limiting the accessibility of the physiological solution within them. The average values of plateau Cu concentrations, ${\left[\text{Cu}\right]}^{0}$, and kinetic constants for all samples are summarized in Table 3.

### 2.5. Antimicrobial Tests

The antimicrobial properties of freshly prepared Cu-polyurethane composites were evaluated in preliminary tests on three target microorganisms selected based on microorganisms characteristics (Gram-positive, Gram-negative, yeasts) to demonstrate the broad-spectrum of NP antimicrobial activity. The suitable dilution tested was obtained taking into account the pathogenic role of microorganisms (mainly *S. aureus* and *E. coli*), the spread and persistence of *S. aureus* on the surfaces, the assessment of *E. coli* as hygiene requirement, and the environment ubiquity of *K. marxianus*. Culture broths were diluted by different factors, based on the different characteristics of the microorganisms (10^7^ for *S. aureus*, 10^5^ for *E. coli* and 10^3^ for *K. marxianus*), and then they were left in contact for fixed times with different samples in order to discriminate the biocidal/biostatic effects of pristine PU foams, electrolytic solution, and CuNP-treated materials. In any sample, after a contact time of 24 h, the residual microorganism growth was quantified by counting the number of colony forming units (CFU). The results are reported in Table 4. As expected, blank experiments on pristine PU foams did not show any biostatic action, whereas control samples treated with 0.1 M TOAC solution in Acetonitrile/tetrahydrofuran (ACN/THF) 1/3_v/v_ mixture produced a strong inhibition effect against *S. aureus*, causing a complete inhibition of the bacterial growth. This is a reasonable result, since TOAC salt belongs to the class of common quaternary ammonium disinfectants. Using Cu-modified polyurethanes induced a marked growth inhibition even in the case of microorganisms such as *E. coli*, that did not show any sensitivity to TOAC-impregnated PU. Finally, comparing the activity of composites treated by colloidal dilutions of 1:100 and 1:1000, it can be concluded that a higher CuNP loading was generally correlated to a higher concentration of released ions, hence in an increased inhibition of colony growth. In some experiments apparent discrepancies between ionic release and bioactivity were observed and attributed to limited reproducibility of the investigated biological systems.

## 3. Materials and Methods

### 3.1. Materials

Copper (0.5 mm thick, 99.99+%) and platinum sheets (0.25 mm thick, 99.999%) were purchased from Goodfellow Ltd. (Cambridge, UK) and cut into 2 × 1 cm pieces. Acetonitrile (ACN, anhydrous, 99.8%), tetrahydrofuran (THF, anhydrous, ≥99.9%, inhibitor-free), and tetraoctylammonium chloride (TOAC, AT reagent, ≥97.0%), were purchased from Sigma Aldrich (Milan, Italy). Aluminum Oxide (purum p.a., 99.7%), for the mechanical polishing of metallic sheets, was from Fluka Chemicals (Milan, Italy). Polyurethane foams were obtained from the industrial partner ME.RES. *Meridionale Resine* S.r.L (Avellino, Italy).

*Escherichia coli* ATCC 25922, *Staphylococcus aureus* FDA 209P (MSSA, with methicillin resistance 0.125 µg·mL^−1^), and *Kluyveromyces marxianus* CBS 608, selected as target microorganisms for biological tests, were obtained from BioMérieux Italia S.p.A. (Florence, Italy), and reconstituted in nutrient broth (Agar Oxoid), purchased from Bio-Chemia (Bari, Italy), as the Plate Count Agar culture medium.

### 3.2. Electrochemical Synthesis of CuNPs

The electrochemical synthesis of CuNPs was carried out in a three-electrode cell equipped with a Cu working electrode and an Ag/AgNO_3_ 0.1 M in ACN reference electrode. The counter electrode was a Pt sheet. The electrolytic solution was composed of 0.1 M TOAC dissolved in an ACN/THF 1/3 mixture. The electrosynthesis was carried out in nitrogen atmosphere, potentiostatically, (+1.5 V vs. reference) for 6 h, under vigorous stirring at room temperature, with a CH1140b potentiostat-galvanostat (CH Instruments, Austin, TX, USA). Other electrochemical parameters such as electrodes pretreatment, electrolysis cell, process details, etc. were similar to what reported in previous works [61,62,63].

### 3.3. Modification of Industrial Polyurethane Foams with CuNPs

Cubic samples, of approximately 1 cm^3^, and weighing about 100 mg, were obtained cutting the as-received materials.

After ascertaining the resistance of each sample towards the solvents used for the electrosynthesis, each material was treated with CuNPs by immersion in 5 mL of diluted colloids for 30 min. After this time, each sample was wrung out and left to dry in air for 2 h. Typically, dilution ratios of CuNP stock solution equal to 1:100 and 1:1000 were used.

### 3.4. Morphological and Spectroscopic Characterization

Colloidal CuNPs diluted in the ratio 1:10 were sonicated for 30 min, before Transmission Electron Microscopy (TEM) analysis, in order to prevent possible aggregation. TEM microscopy was performed with a FEI Tecnai 12 instrument (Hillsboro, OR, USA, high tension: 120 kV; filament: W), by dropping 10 µL on carbon-coated Cu grids (300 mesh, TAAB Laboratories Equipment Ltd., Aldermaston, UK). The microscope was calibrated using the S106 Cross Grating (2160 lines/mm, 3.05 mm) supplied by Agar Scientific (Stansted, UK). Alignment was checked by using factory settings and routines. Astigmatism was adjusted by means of fast Fourier transform processing. Size distribution of metal clusters was evaluated using ImageJ software [64]. Treated and pristine polyurethane materials were characterized by means of X-ray photoelectron spectroscopy (XPS), using a Thermo Fisher Scientific Theta Probe Spectrometer (Waltham, MA, USA). A monochromatized AlKα source was used, with a beam spot diameter of 300 μm. Samples were mounted onto the sample holder by means of a carbon double-side copper tape (Agar Scientific, Stansted, UK). All XPS measurements were performed in constant analyzer energy (CAE) mode. Survey and high-resolution spectra (C1s, O1s, Si2p, Cu2p_3/2_, N1s, Ca2p and Cl2p) were acquired at a pass energy of 150 and 100 eV, respectively, and with a step size of 1.0 and 0.1 eV, respectively. Detailed spectra processing was performed by commercial Thermo Avantage software (v. 4.75© 1999–2010 Thermo Fisher Scientific, Waltham, MA, USA). Curve-fitting analysis was applied to the Cu2p_3/2_ high-resolution spectra, in order to assess Cu chemical state. Surface atomic percentages were determined after Shirley background removal, using *Scofield* sensitivity factors. The same peak lineshape parameters (Gaussian/Lorentzian ratio and full width at half maximum) values were employed for the curve fitting of components belonging to the same high-resolution spectrum. Spectra were corrected for charge compensation effects by offsetting the binding energy relative to the aliphatic component of the C1s spectrum, which was set to 284.8 eV.

### 3.5. Kinetics of Copper Release in Aqueous Solution by Electro-Thermal Atomic Absorption Spectroscopy (ETAAS)

Copper release from CuNP-modified polyurethane foams was evaluated by electro-thermal atomic absorption spectroscopy (ETAAS). Cu quantification was achieved by means of a calibration curve obtained by the analysis, carried out in triplicate and in random sequence, of standard solutions at known Cu concentration, prepared by subsequent dilution of a stock Cu commercial solution (Fluka, Milan, Italy, Copper Standard for AAS TraceCERT^®^, 1000 mg/L Cu in nitric acid). Each PU sample was immersed in a Pyrex bottle containing 25 mL of contact solution. The latter was obtained as a 1:1 mixture of a 0.85% *w*/*w* NaCl aqueous solution (Fluka, purity ≥99.5%) with phosphate buffer (PBS) K_2_HPO_4_/NaH_2_PO_4_ with known pH and ionic strength (6.4 and 0.1, respectively). At fixed times (10 min, 1 h, 2 h, 4 h, 7 h, 10 h and 24 h), 200 µL of this solution were taken from each vessel. These samples were appropriately diluted and acidified by 0.7% HNO_3_ solution; if needed, additional dilution was applied in order to keep the measured Cu concentration within the linearity interval of the calibration curve. Samples were analyzed by a Perkin-Elmer dual-beam spectrophotometer, model 460 (Milan, Italy), using electro-thermal atomization in graphite furnace. Source was a hollow-cathode lamp (absorption line at 324.7 nm). The signal acquisition mode provided an automatic background correction by a deuterium lamp, in order to eliminate possible matrix absorption overlapping with the Cu absorption band. The entire kinetic experiment was carried out at 25 °C. The measured data were fitted by a first order kinetic Equation (1), where ${\left[\text{Cu}\right]}^{0}$ represented the amount of copper ions immediately dissolved (and therefore immediately available) in solution, ${\left[\text{Cu}\right]}^{\text{max}}$ was the maximum copper concentration in solution reached within 24 h, and k was the release kinetic constant. Data analysis was carried out using SigmaPlot^®^ 12.0 software (Systat Software, San Jose, CA, USA).

$$\left[\text{Cu}\right]={\left[\text{Cu}\right]}^{0}+{\left[\text{Cu}\right]}^{\text{max}}\left(1-e^{-\text{kt}}\right)$$ (1)

The possible Cu release from both vessels/glassware and the untreated polyurethane foams was also assayed; and the resulting Cu concentrations were all below the limit of quantification (LOQ).

### 3.6. Antimicrobial Tests

*Escherichia coli* ATCC 25922, *Staphylococcus aureus* FDA 209P (MSSA, with methicillin resistance 0.125 µg/mL), and *Kluyveromyces marxianus* CBS 608 were selected as target microorganisms. Aliquots of the different lyophilized microorganisms were reconstituted in 0.9% NaCl solution, added to 20 mL of sterile nutrient broth (Oxoid) and incubated for 24 h at the optimal temperature for microbial growth, which was 37 °C for *Staphylococcus aureus*, 42 °C for *Escherichia coli*, and 28 °C for *Kluyveromyces marxianus*. Each sample was then diluted 10^5^ times by saline solution (pH = 6.4, [Cl^−^] = 0.15 M). 5 mL of each culture broth were left in contact with each type of CuNP treated foams, and incubated for 24 h, at the respective optimal growth temperatures. Untreated samples were used as controls. After the selected incubation times, 1 mL of culture broth was taken from each vessel and inoculated in a Petri dish containing nutrient agar. After 24 h, bacterial colony count was performed on each Petri dish, in order to assess the entity of bacterial growth inhibition exerted by treated polyurethane foams.

## 4. Conclusions

Sacrificial-anode electrochemical synthesis of TOAC-stabilized CuNPs was used to modify industrial polyurethane foams. TEM and XPS analyses on Cu-nanocolloids demonstrated that the surfactant employed was capable of stabilizing freshly dispersed CuNPs from both the morphological and the chemical point of view. XPS measurements on copper-polyurethane composites revealed that a simple impregnation protocol employing diluted colloids was effective in modifying industrial polyurethane foams with CuNPs. These data, along with copper ion release measurements, showed that the final copper surface availability, along with the release of antibacterial ions in physiological solution, could be tuned just by changing the CuNP concentration in the impregnation baths. Foam pore size and aging resulted to affect the ion release to a minor extent, although samples stored for two months were demonstrated to be still very active in releasing bioactive copper ions. Biological tests showed that the proposed nano-functionalized materials exert a marked inhibitory effect on the growth of different target microorganisms such as *S. aureus*, *E. coli* and *K. marxianus*.

## Figures and Tables

**Table 1:** Surface elemental composition estimated by X-ray photoelectron spectroscopy (XPS) of fresh samples A and B, treated with CuNPs. Error is expressed as the larger value between the error associated to a single quantification (0.2% for copper, 0.5% for other elements) and one standard deviation, calculated on at least three replicate analyses. Data about pristine samples are reported for comparison.

Element	Sample A			Sample B		
	Pristine	PU/CuNPs (1:100)	PU/CuNPs (1:1000)	Pristine	PU/CuNPs (1:100)	PU/CuNPs (1:1000)
**Cu**	<0.2%	1.3 ± 0.2	0.8 ± 0.2	<0.2%	0.5 ± 0.2	0.3 ± 0.2
**C**	73.7 ± 0.5	76.9 ± 0.5	79 ± 3	72.6 ± 0.5	68.4 ± 0.5	67.5 ± 0.5
**N**	1.6 ± 0.5	1.7 ± 0.5	1.3 ± 0.5	1.6 ± 0.5	1.6 ± 0.5	2.8 ± 0.5
**O**	23.3 ± 0.5	18.5 ± 0.5	18 ± 3	20.7 ± 0.5	24.4 ± 0.5	23.7 ± 0.5
**Si**	1.4 ± 0.5	1.6 ± 0.5	0.9 ± 0.5	4.5 ± 0.5	5.1 ± 0.5	5.7 ± 0.5
**Cl**	–	<0.5	<0.5	–	<0.5	<0.5
**Ca**	–	–	–	0.6 ± 0.5	<0.5	<0.5

**Table 2:** Attributions of C1s chemical environments identified on type A pristine and Cu-modified PU foams; relative abundance % of each signal component is reported for comparison. Error is expressed as one standard deviation, calculated on at least three replicate analyses.

Sample	BE (eV)	Attribution	Relative Abundance %
Pristine	284.8 ± 0.1	C–C	43 ± 2
	286.4 ± 0.2	C–O, C–N	55.8 ± 1.3
	289.0 ± 0.2	HN–C=O	1.2 ± 0.8
PU/CuNPs (1:100)	284.8 ± 0.1	C–C	55 ± 3
	286.4 ± 0.2	C–O, C–N	42 ± 2
	288.8 ± 0.2	HN–C=O	3.0 ± 1.3

**Table 3:** Average values of plateau Cu concentrations and kinetic constants for samples A and B. Data about fresh and aged samples are reported. The error is expressed as the largest value between the standard deviation relevant to the repeated measurements and the error associated to individual quantifications.

Sample	CuNPs Dilution	Plateau [Cu]/ppb		Kinetic Constant/h^−1^		[Cu]^0^/ppb	
		Fresh	Aged	Fresh	Aged	Fresh	Aged
Sample A	1:100	1200 ± 90	670 ± 40	0.20 ± 0.04	0.20 ± 0.04	0	40 ± 30
	1:1000	300 ± 40	330 ± 20	0.8 ± 0.3	0.20 ± 0.04	50 ± 30	0
Sample B	1:100	1100 ± 200	550 ± 40	0.2 ± 0.1	0.40 ± 0.09	300 ± 100	40 ± 30
	1:1000	260 ± 30	270 ± 20	0.5 ± 0.1	0.4 ± 0.1	30 ± 20	20 ± 20

**Table 4:** Number of colony forming units (CFU) for the three target microorganisms, exposed to different samples for 24 h as described in the experimental section. Error on CFU counts is ±5 in the last digit.

Sample		*S. aureus*/CFU	*E. coli*/CFU	*K. marxianus*/CFU
Sample A	PU	U ^a^	U	U
	PU + 0.1 M TOAC solution	0	U	U
	PU + 1:1000 CuNPs	0	0	30
	PU + 1:100 CuNPs	0	0	25
Sample B	PU	U	U	U
	PU + 0.1 M TOAC solution	2	U	U
	PU + 1:1000 CuNPs	U	72	U
	PU + 1:100 CuNPs	0	0	U

^a^ U = Uncountable.

## Abbreviations

The following abbreviations are used in this manuscript:

BE	binding energy
CFU	colony forming unit
CNT	carbon nanotube
CuNP	copper nanoparticle
ETAAS	electro-thermal atomic absorption spectroscopy
PBS	phosphate buffer saline
PU	polyurethane
SAE	sacrificial anode electrolysis
TEM	transmission electron microscopy
TOAC	tetraoctylammonium chloride
XPS	X-ray photoelectron spectroscopy
